# A Novel Method of CD31-Combined ABO Carbohydrate Antigen Microarray Predicts Acute Antibody-Mediated Rejection in ABO-Incompatible Kidney Transplantation

**DOI:** 10.3389/ti.2022.10248

**Published:** 2022-03-23

**Authors:** Masayuki Tasaki, Hiroaki Tateno, Takashi Sato, Azusa Tomioka, Hiroyuki Kaji, Hisashi Narimatsu, Kazuhide Saito, Yuki Nakagawa, Toshinari Aoki, Masami Kamimura, Takashi Ushiki, Manabu Okada, Yuko Miwa, Kiyohiko Hotta, Yutaka Yoshida, Kota Takahashi, Yoshihiko Tomita

**Affiliations:** ^1^ Division of Urology, Department of Regenerative and Transplant Medicine, Graduate School of Medical and Dental Sciences, Niigata University, Niigata, Japan; ^2^ Cellular and Molecular Biotechnology Research Institute, National Institute of Advanced Industrial Science and Technology (AIST), Tsukuba, Japan; ^3^ Department of Urology, Juntendo University Graduate School of Medicine, Tokyo, Japan; ^4^ Department of Transfusion Medicine, Cell Therapy and Regenerative Medicine, Niigata University Medical and Dental Hospital, Niigata, Japan; ^5^ Department of Transplant Surgery, Nagoya Daini Red Cross Hospital, Nagoya, Japan; ^6^ Department of Kidney Disease and Transplant Immunology, Aichi Medical University School of Medicine, Nagoya, Japan; ^7^ Department of Urology, Hokkaido University Hospital, Sapporo, Japan; ^8^ Department of Structural Pathology, Kidney Research Center, Graduate School of Medical and Dental Sciences, Niigata University, Niigata, Japan; ^9^ Takahashi Memorial Medical Institute, Tokyo, Japan

**Keywords:** antibody-mediated rejection, ABO-incompatible kidney transplantation, antibody titer, CD31, microarray

## Abstract

Isohemagglutinin assays employing red blood cells (RBCs) are the most common assays used to measure antibody titer in ABO-incompatible kidney transplantation (ABOi KTx). However, ABO antigens expressed on RBCs are not identical to those of kidney and antibody titers do not always correlate with clinical outcome. We previously reported that CD31 was the main protein linked to ABO antigens on kidney endothelial cells (KECs), which was different from those on RBCs. We developed a new method to measure antibody titer using a microarray of recombinant CD31 (rCD31) linked to ABO antigens (CD31-ABO microarray). Mass spectrometry analysis suggested that rCD31 and native CD31 purified from human kidney had similar ABO glycan. To confirm clinical use of CD31-ABO microarray, a total of 252 plasma samples including volunteers, hemodialysis patients, and transplant recipients were examined. In transplant recipients, any initial IgG or IgM antibody intensity >30,000 against the donor blood type in the CD31-ABO microarray showed higher sensitivity, specificity, positive predictive value, and negative predictive value of AABMR, compared to isohemagglutinin assays. Use of a CD31-ABO microarray to determine antibody titer specifically against ABO antigens expressed on KECs will contribute to precisely predicting AABMR or preventing over immunosuppression following ABOi KTx.

## Graphical Abstract

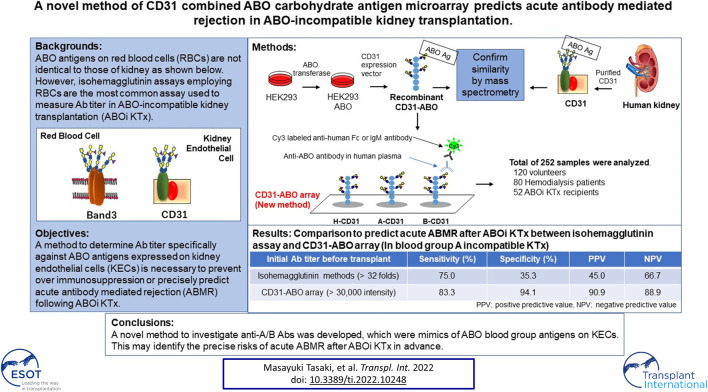

## Introduction

In most countries, a paired donation program to circumvent the immunological challenge of ABO incompatibility is precluded by law. Therefore, a kidney transplant candidate with an ABO-incompatible (ABOi) living donor has a valuable option to wait for a deceased ABO-compatible donor with long-term dialysis therapy. Recent cohort studies have shown no significant difference in patient and graft survival in ABOi kidney transplantation (KTx) compared to ABO-compatible (ABOc) KTx (1–5). However, recent meta-analysis has shown lower patient and graft survival in ABOi KTx than ABOc KTx (6, 7). In ABOi KTx, over immunosuppression, leading to life-threatening infections, may cause lower patient survival (6, 7). In addition, acute antibody-mediated rejection (AABMR), due to anti-A or -B antibodies (Abs), contributes to lower graft survival (6, 7). Ab titers against donor blood group antigen may be an AABMR predictor following ABOi KTx, and tailored desensitization therapy according to Ab titer may avoid over immunosuppression (8). However, an acceptable Ab titer against donor blood group antigen to prevent AABMR has not been defined in ABOi KTx. In addition, the desensitization therapy protocol varies from institution to institution and the method to measure Ab titer is not unified.

Technological advances in HLA laboratory testing undoubtedly improved the sensitivity and specificity of HLA Ab assessment. Multiple methodologies such as complement-depending cytotoxicity test, flow cytometry, and Luminex-based technology can be available for HLA Abs test. The understanding of complement (C1q and C3d, etc) fixing Abs and IgG subclass in HLA Abs has become widespread. In contrast, Ab test against ABO antigens in ABOi organ transplantation is still primitive. Isohemagglutinin assays employing red blood cells (RBCs) are the most common assay used to measure Ab titer in ABOi KTx. However, ABO blood group antigens expressed on RBCs are not identical to those of the kidney due to different proteins linked to ABO carbohydrate antigens (9). Ab epitopes against ABO blood group antigens may differ between RBCs and endothelial cells (10). In some cases, Ab titers do not correlate with clinical outcome; AABMR does not occur in some patients with high Ab titers, and vice versa (11–13). A method to determine Ab titer specifically against ABO blood group antigens expressed on kidney endothelial cells (KECs) is necessary to prevent over immunosuppression or precisely predict AABMR following ABOi KTx.

Pecam1 (CD31) is the most abundant protein linked to ABO blood group antigens on KECs, which is different from Band3 mainly expressed on RBCs (9). Here, a new method was developed to measure Ab titer using a microarray of CD31 linked to ABO carbohydrate antigens (CD31-ABO microarray) which is a mimic of ABO blood group antigens on KECs. This novel method may precisely predict AABMR following ABOi KTx.

## Materials and Methods

### Sample and Data Collection

A total of 252 plasma samples were collected. Volunteers (*n* = 120) donated blood samples at the Japan Red Cross blood center. Approval for this study was obtained from the Japanese Red Cross Institutional Review Board (authorization number 28J0001). Samples were donated without personal identifiers. The only available demographic factor was ABO blood type for these samples. Other plasma samples were collected from patients undergoing hemodialysis (*n* = 80) and recipients (*n* = 52) who received ABOi KTx at the Niigata University Medical and Dental Hospital, Nagoya Daini Red Cross Hospital, and Hokkaido University Hospital, Japan. All participants in this study were Japanese. All transplantations were living-donor KTx. Clinical and laboratory information was extracted from electronic databases and patients’ medical records. Transplant recipients were divided into two groups: patients without AABMR (-) and with AABMR (+) due to anti-A or B Abs after ABOi KTx. The study was performed in accordance with the guidelines of the Declaration of Helsinki, subsequent to approval from the hospital’s Institutional Ethical committee (authorization number 2018-0311).

### Anti-ABO Ab Isohemagglutinin Titers

Titration of anti-A and anti-B Abs were performed using the test tube method, as described in detail in the Supplementary Methods.

### Immunosuppression for ABOi KTx

Immunosuppression therapy was performed according to the protocol at each institution, as described in detail in the Supplementary Methods. Plasma exchange or double-filtration plasmapheresis was performed before ABOi KTx to decrease Ab titers. Splenectomy was performed on the day of ABOi KTx before 2003, and rituximab was used after 2004, instead of splenectomy. Calcineurin inhibitors, methylprednisolone, mycophenolate mofetil, and basiliximab were given for induction therapy, with the exception of a few cases.

### AABMR Diagnosis

There were no recipients who had donor human leukocyte antigen (HLA) specific performed Abs in this cohort. Whenever a rejection was clinically suspected, an episode biopsy was performed. A rejection diagnosis was made by the pathologist at each institution. AABMR due to anti-A or B Abs was diagnosed using pathological findings of ABMR (Banff19) when anti-donor HLA Abs were not detected at the time of rejection.

### Preparation of Recombinant CD31 Containing ABO Carbohydrate Antigens

**FIGURE 1:**
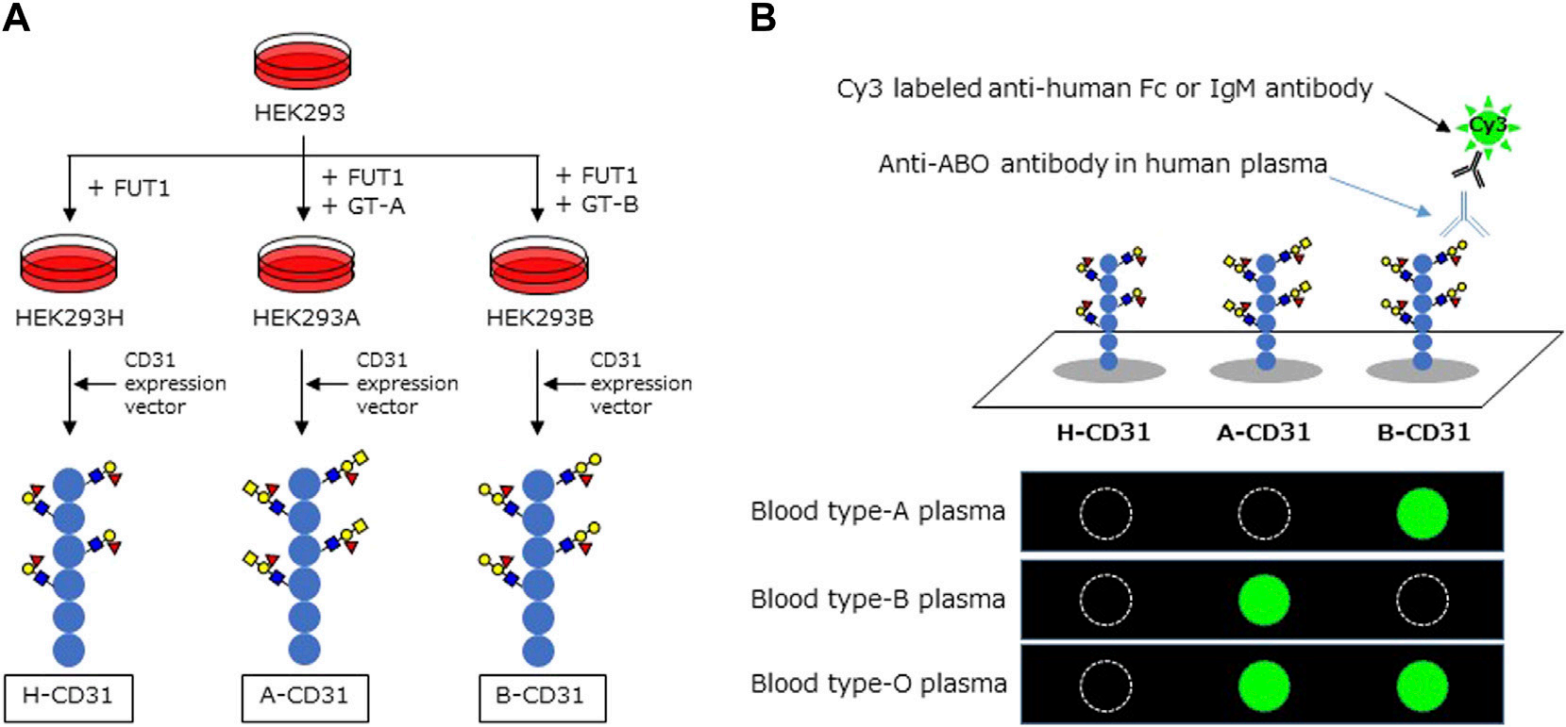
Schema of CD31 linked to ABO carbohydrate antigen microarray. **(A)** The development of recombinant CD31 proteins containing ABO carbohydrate antigen. **(B)** Analyzing anti-A and B antibodies levels in CD31-ABO microarray. HEK: human embryonic kidney, FUT1: α1,2 fucosyltransferase, GT-A: α1,3 *N*-acetylgalactosaminyltransferase, GT-B: α1,3 galactosyltransferase.

Recombinant CD31 proteins (rCD31) containing ABO carbohydrate antigens were produced in glycogene-modified human embryonic kidney (HEK293) cells. H-type glycan-expressing cells were established by overexpression of α1,2 fucosyltransferase (*FUT1*) into HEK293 cells; the resulting cells were designated HEK293H. A-type glycan- and B-type glycan-expressing cells were established by overexpression of α1,3 *N*-acetylgalactosaminyltransferase (*GT-A*), and α1,3 galactosyltransferase (*GT-B*) into HEK293H, respectively, and designated HEK293A and HEK293B. The cDNA encoding the extracellular domain of CD31 was amplified by polymerase chain reaction using the following primers: Forward: 5ʹ-aag​ctt​cag​gAT​GCA​GCC​GAG​GTG​GGC​CCA-3ʹ, including the HindIII site and Reverse: 5ʹ-gcg​gcc​gcT​TCT​TCC​ATG​GGG​CAA​GAA​TGA-3ʹ, including the NotI site, and cDNA derived from human umbilical vein endothelial cells as a template. An approximately 1.8 kb DNA fragment was amplified and subcloned into the pCRII-blunt vector (Life Technologies). After confirmation of the correct sequence using a Genetic Analyzer 3130xl (Applied Biosystems), the HindIII and NotI fragment was inserted into the pcDNA3.1n-F expression vector, which was modified from pcDNA3.1n(+) (Life Technologies) by introducing the sequence encoding DYKDDDDK and a termination codon. The resulting plasmid, designated pcDNA3.1n-CD31-F, was transfected into HEK293H, HEK293A, and HEK293B cells using Lipofectamine LTX (Life Technologies), to produce rCD31 with a FLAG tag at the C-terminus in culture medium. After 48–72 h incubation at 37°C, each medium was collected and rCD31 was purified using an anti-FLAG M2 agarose affinity gel (Sigma-Aldrich). The culture medium (300 ml) was mixed with 500 μL suspension of anti-FLAG M2 agarose affinity gel and rotated slowly at 4°C for several hours. After centrifugation, the gel was washed 2–5× with PBS containing 0.01% Tween-20 and rCD31 was eluted from the affinity gel using a FLAG peptide (Sigma-Aldrich). The protein concentration of purified rCD31 was determined using a NanoDrop LITE spectrophotometer (Thermo Scientific) and was designated H-CD31, A-CD31, and B-CD31, respectively (Figure 1A).

### Preparation of CD31 Proteins From Human Kidneys

Kidney tissues were obtained from patients, with their informed consent, who underwent surgical nephrectomy due to renal carcinoma at the Niigata University Medical and Dental Hospital. Proteins were extracted from normal kidney cortices of patients with different ABO blood types and CD31 proteins were purified, as described in detail in the supplementary materials and methods. Protein extracts were incubated with Dynabeads protein G (VERITAS) pre-bound to anti-CD31 Ab (Santa Cruz Biotechnology). Dynabeads were thoroughly washed with lysis solution and eluted with sodium dodecyl sulfate (SDS) sample buffer. The eluates with SDS sample buffer were separated by SDS-polyacrylamide gel electrophoresis (PAGE). SDS-PAGE gel pieces containing CD31 protein with molecular mass approximately 130 kDa were excised for mass spectrometry (MS).

### Mass Spectrometry Analyses of CD31 Glycopeptides

Identification of *N*-glycosylated Asn sites and site-specific analysis of glycan compositions and structures for both the rCD31 and CD31 proteins from human kidneys were conducted using the IGOT (14) and Glyco-RIDGE (15) methods, respectively. CD31 proteins were digested with Lysyl endopeptidase and trypsin. The digests were separated by hydrophilic interaction chromatography on Amide-80 column (TOSOH) to collect glycopeptides. An aliquot of glycopeptide was treated with peptide-*N*-glycanase in H_2_^18^O to remove *N*-glycan and label the glycosylated Asn residues with ^18^O for identification of *N*-glycosylated sites (IGOT method). Another aliquot of glycopeptides was heated in 0.1% trifluoroacetic acid to remove sialic acids. The deglycosylated or desialylated glycopeptides were analyzed using a nano-flow liquid chromatography-coupled Orbitrap Fusion Tribrid mass spectrometer (Thermo Scientific). Desialylated glycopeptides were analyzed for their site-specific glycan compositions and partial structure using the Glyco-RIDGE method. For more experimental details, refer to the supplementary methods.

### Microarray of CD31 Linked to ABO Carbohydrate Antigens (CD31-ABO Microarray)

The CD31-ABO microarray was produced as described previously (detail in the Supplementary Methods) (16). rCD31 containing ABO carbohydrate antigens (H-CD31, A-CD31, and B-CD31) were dissolved at a concentration of 0.1 mg/ml in a spotting solution (Matsunami Glass) and spotted onto epoxysilane-coated glass slides (Schott) in triplicate using a non-contact microarray printing robot (Microsys4000, Genomic Solutions). The glass slides were incubated at 25°C overnight to allow immobilization, washed with probing buffer, and incubated with the blocking reagent at 20°C for 1 h. Finally, glass slides were washed with TBS containing 0.02% NaN3 and stored at 4°C until use.

Human plasma (80 µL/well) was diluted 100-fold with probing buffer and incubated with the CD31-ABO microarray at 20°C overnight. After washing twice with 100 µL/well probing buffer, 1 μg/ml Cy3-conjugated goat anti-human Fc (Jackson ImmunoResearch: 109-165-098) or Cy3-conjugated goat anti-human IgM (Jackson ImmunoResearch: 109-165-043) were added and incubated at 20°C for 1 h. Fluorescence images were acquired using an evanescent field-activated fluorescence scanner Bio-REX Scan200 (Rexxam). The fluorescence signal of each spot was quantified using Array Pro Analyzer version 4.5 (Media Cybernetics), and background values were subtracted. Background values were obtained from an area without immobilized samples (Figure 1B). Anti-A and B Ab levels (relative intensity) were calculated as the subtraction of the H-CD31 reaction from the A-CD31 or B-CD31 reaction in each sample.

### Statistical Analysis

The continuous variables are expressed as the mean ± standard deviation, and the categorical variables are expressed as N and percentages. A Mann-Whitney *U*-test or student’s *t*-test was used to compare two groups of continuous variables, and a chi-square test was used to compare categorical data. The diagnostic potential of the CD31-ABO microarray was determined by calculating the receiver operating characteristic (ROC) curve plotted to evaluate the sensitivity and specificity for predicting AABMR after ABOi KTx. The sensitivity, specificity, positive predictive value (PPV), and negative predictive value (NPV) were used to investigate its accuracy as a diagnostic tool for AABMR after ABOi KTx.

## Results

### ABO Glycan Analysis of rCD31 and Human Kidney CD31 by MS

We successfully produced rCD31-contained ABH glycans by using HEK293 cells *in vitro* and used them for microarray. We needed to know whether rCD31 in this microarray had similar ABH glycans to those of native human kidney. Using the Glyco-RIDGE method, glycan compositions of two core glycopeptides (VLENSTK, including Asn-453, and EGKPFYQMTSNATQAFWTK, including Asn-551) derived from rCD31 proteins purified from culture media of HEK193H, HEK293A, and HEK293B cells were assigned and compared (Supplementary Figures S1, S2). The glycan composition of each signal is shown in Supplementary Figure S1 as XYZ corresponding to the numbers of Hex, HexNAc, and dHex (Fucose) on the tri-mannosyl core (Man3GlcNAc2 = 000). The compositions containing multiple fucoses are shown in blue. These glycopeptides are presumed to have blood type glycans, since one fucose at least is attached on non-reducing terminus. Characteristic or increased compositions in blood type A or B are shown in each spectrum with triangles. In Supplementary Figure S2, generation of blood type glycans is suggested clearly. For example, 232, significant in type H, seemed to be shifting to 242 of type A, and to 332 of type B, suggesting the generation of type A and type B antigens, respectively. CD31 prepared by immunoprecipitation from the normal parts of human kidney extract followed by SDS-PAGE was analyzed by the same way as rCD31 (Supplementary Figure S3). Accumulated spectra of the glycopeptide (VLENSTK containing Asn-453) of each blood type are compared. Signals assigned to the CD31 glycopeptide are marked with their compositions and the MS/MS spectra of signals marked with red triangle were compared (Supplementary Figures S4–S6). All MS/MS spectra show the presence of core fucose and glycan-derived signals such as Hex(1)HexNAc(1)Fuc(1), Hex(1)HexNAc(2)Fuc(1), and Hex(2)HexNAc(1)Fuc(1), suggesting the presence of blood group antigens of blood type H, A, and B (for more details see “Supplementary Results”). Taken together, rCD31 used for the CD31-ABO microarray had the glycopeptide (VLENSTK) conjugated to the blood group H, A, and B glycan, and CD31 derived from human kidney had the same glycopeptide, which was strongly suggested to have blood group H, A, and B glycans.

### Anti-A and B Abs in Volunteers and Hemodialysis Populations

The results of Ab levels measured using the CD31-ABO microarray are shown in Tables 1, 2. These microarrays specifically detected anti-A and anti-B Abs. Anti-A and B Ab levels were not significantly different between volunteers and hemodialysis populations. Both anti-A and B IgG Ab levels were significantly higher in the type O population than those in the type B and A populations, respectively (*p* < 0.01). However, anti-A and B IgM Ab levels were not significantly different between the type O and type B, and type O and type A populations, respectively. We analyzed the same samples by using the isohemagglutinin method (Tables 1, 2), showing a similar trend as CD31-ABO microarray.

**TABLE 1:** The results of anti-A antibodies in A-CD31 microarray compared to isohemagglutinin assay, median (range).

		Anti-A Ab (IgG)			Anti-A Ab (IgM)		
		Healthy volunteers (*n* = 120)	Hemodialysis patients (*n* = 80)	*p*-value	Healthy volunteers (*n* = 120)	Hemodialysis patients (*n* = 80)	*p*-value
O	microarray	58199 (3235–62235)	62146 (5810–65535)	0.596	14666 (1572–59954)	12344 (1819–29384)	0.268
	isohemagglutinin	48 (4–512)	64 (8–1024)	0.344	24 (4–64)	16 (4–64)	0.128
A	microarray	0 (0–224)	0 (0–1548)	0.382	0 (0–0)	0 (0–445)	0.314
	isohemagglutinin	0 (0)	0 (0)	N/A	0 (0)	0 (0)	N/A
B	microarray	4924 (0–22223)	6687.5 (1436–54723)	0.243	12967 (489–55322)	17157.5 (2859–59642)	0.403
	isohemagglutinin	1 (1–16)	2 (1–32)	0.101	16 (4–128)	8 (4–64)	0.639
AB	microarray	0 (0–891)	0 (0–98)	0.571	0 (0–878)	0 (0–0)	0.791
	isohemagglutinin	0 (0)	0 (0)	N/A	0 (0)	0 (0)	N/A

N/A, not applicable.

**TABLE 2:** The results of anti-B antibodies in B-CD31 microarray compared to isohemagglutinin assay, median (range).

		Anti-B Ab (IgG)			Anti-B Ab (IgM)		
		Healthy volunteers (*n* = 120)	Hemodialysis patients (*n* = 80)	*p*-value	Healthy volunteers (*n* = 120)	Hemodialysis patients (*n* = 80)	*p*-value
O	microarray	32564 (0–65416)	17311 (1677–65244)	0.575	4877 (201–61871)	3466 (341–62727)	0.339
	isohemagglutinin	48 (1–512)	32 (1–512)	0.923	16 (4–64)	16 (2–64)	0.862
A	Microarray	173 (0–7421)	89 (0–10772)	0.883	4346 (0–28812)	4422 (0–31820)	0.634
	isohemagglutinin	1 (1–4)	1 (1–8)	0.164	12 (2–32)	8 (2–64)	0.672
B	Microarray	0 (0–614)	0 (0–1614)	0.791	0 (0–0)	0 (0–2762)	0.134
	isohemagglutinin	0 (0)	0 (0)	N/A	0 (0)	0 (0)	N/A
AB	Microarray	0 (0–1271)	0 (0–1)	0.382	0 (0–2893)	0 (0–572)	0.837
	isohemagglutinin	0 (0)	0 (0)	N/A	0 (0)	0 (0)	N/A

N/A, not applicable.

**FIGURE 2:**
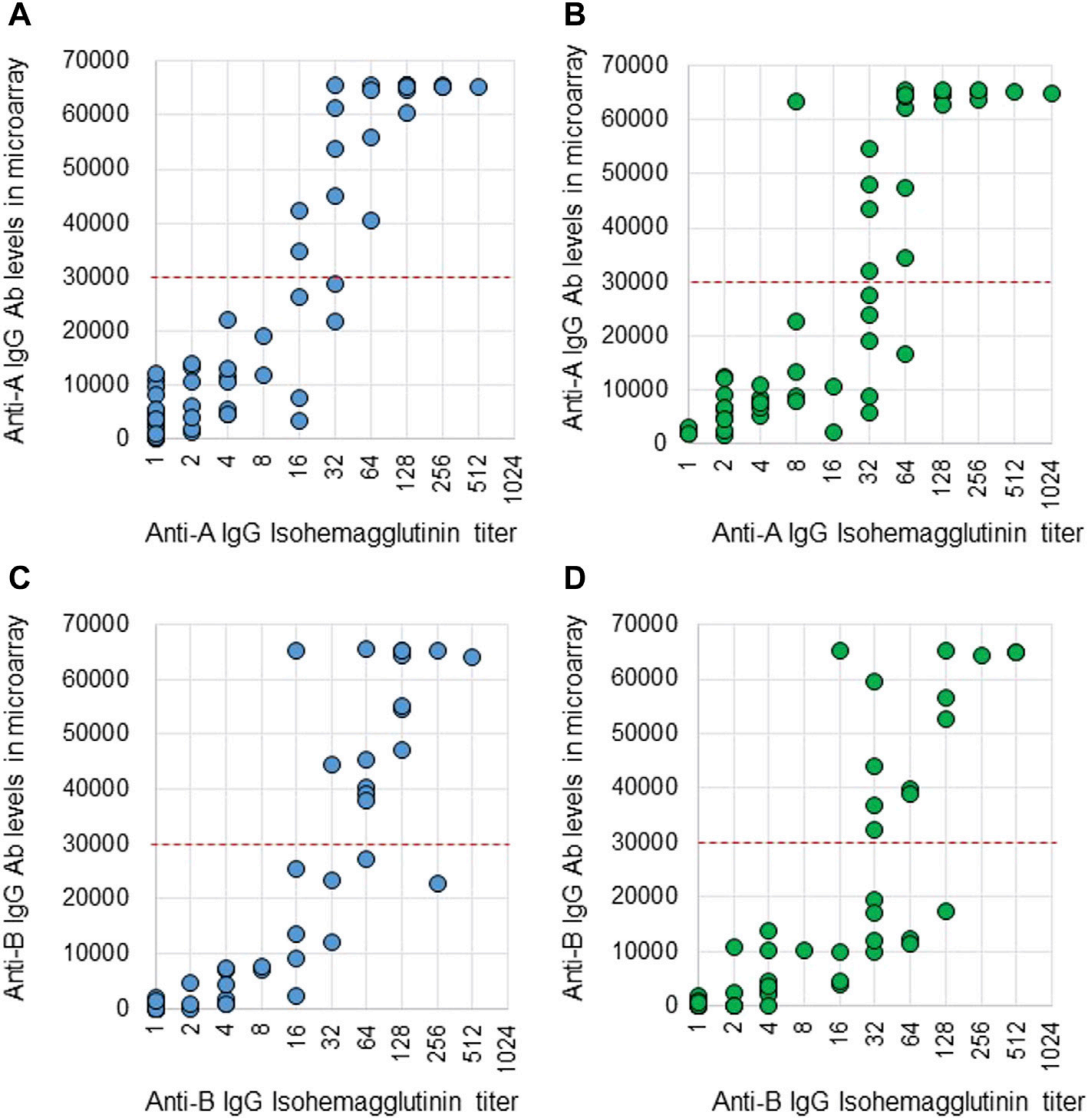
Comparison of anti-A and B IgG Abs between the isohemagglutinin and CD31-ABO microarray methods in volunteers and hemodialysis patients. **(A)** Anti-A IgG Ab in volunteers. **(B)** Anti-A IgG Ab in hemodialysis patients. **(C)** Anti-B IgG Ab in volunteers. **(D)** Anti-B IgG Ab in hemodialysis patients. The x-axis is isohemagglutinin titers on a log2 scale; titer values of zero are displayed as 1 on the graph. The y-axis is antibody level on the CD31-ABO microarray. The red dot-line shows the cut-off levels of the CD31-ABO microarray for predicting AABMR.

**FIGURE 3:**
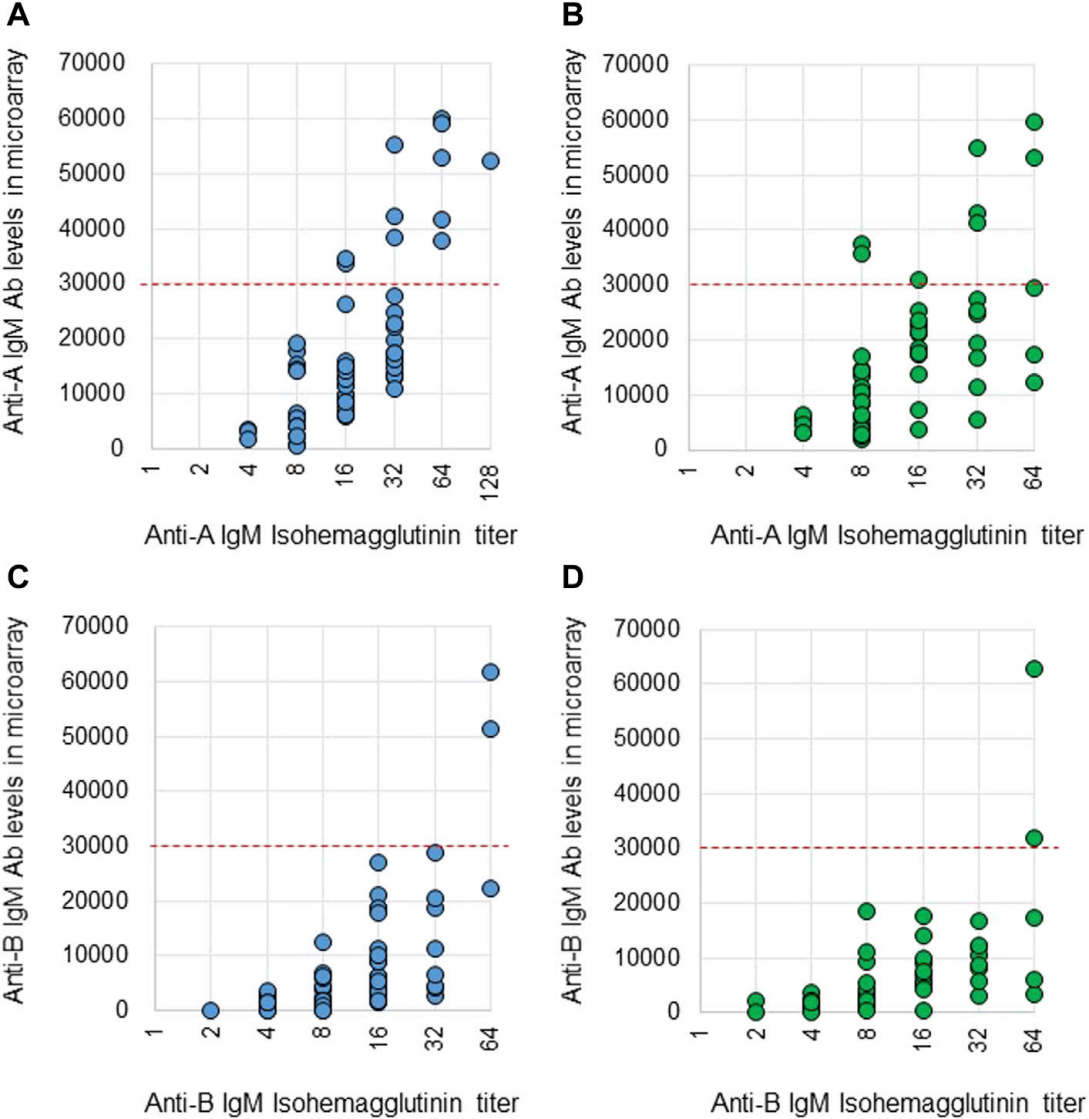
Comparison of anti-A and B IgM Abs between the isohemagglutinin and CD31-ABO microarray methods in volunteers and hemodialysis patients. **(A)** Anti-A IgM Ab in volunteers. **(B)** Anti-A IgM Ab in hemodialysis patients. **(C)** Anti-B IgM Ab in volunteers. **(D)** Anti-B IgM Ab in hemodialysis patients. The x-axis is isohemagglutinin titers on a log2 scale; titer values of zero are displayed as 1 on the graph. The y-axis is antibody level on the CD31-ABO microarray. The red dot-line shows the cut-off levels of the CD31-ABO microarray for predicting AABMR.

Anti-A and B Abs were compared between these two methods. Ab titers in the isohemagglutinin method and Ab levels in the CD31-ABO microarray were roughly correlated in volunteers and the hemodialysis population (Figure 2, 3). However, Ab levels in the CD31-ABO microarray varied even in samples with the same isohemagglutinin titers.

### Patient Characteristics

Table 3 shows the patient characteristics in the two groups divided by the existence of AABMR after ABOi KTx. There were no significant differences, except for Ab removal therapy before ABOi KTx. Ab titers before desensitization therapy and on the day of ABOi KTx against donor blood type measured using the isohemagglutinin method were not significantly different between the two groups (data not shown). The median post-operative day at diagnosis of AABMR was 5 (range; 0–19).

**TABLE 3:** Patients’ demographics and clinic characteristics in ABOi KTx patients.

	ABOi KTx w/o AABMR (*n* = 31)	ABOi KTx with AABMR (*n* = 21)	*p*-value
Male, n (%)	22 (71.0)	13 (61.9)	0.556
Age, y.o, median (range)	44 (23–63)	54.0 (14–76)	0.066
Duration of dialysis (M), median (range)	14 (0–213)	3 (0–119)	0.896
Donor age, y.o, median (range)	55 (30–69)	58 (38–74)	0.111
ABO incompatible transplantation			
*A-incompatible, n (%)*	21 (67.7)	15 (71.4)	0.785
*B-incompatible, n (%)*	7 (22.6)	6 (28.6)	0.842
*AB-incompatible, n (%)*	3 (9.7)	0 (0.0)	0.060
HLA mismatch^a^, mean ± SD	3.2 ± 1.3	3.8 ± 1.5	0.118
Preemptive KTx, n (%)	8 (25.8)	7 (33.3)	0.756
WIT (min), mean ± SD	3.2 ± 1.8	2.7 ± 1.6	0.404
TIT (min), mean ± SD	98.7 ± 50.2	93.6 ± 34.2	0.692
Immunosuppression			
*FK, n (%)*	20 (71.0)	11 (52.4)	0.405
*CyA, n (%)*	11 (29.0)	10 (47.6)	0.405
*MMF, n (%)*	29 (93.5)	20 (95.2)	1.000
*AZ, n (%)*	2 (6.5)	1 (4.8)	1.000
*CPA, n (%)*	0 (0.0)	3 (14.3)	0.060
*Rituximab, n (%)*	28 (90.3)	14 (66.7)	0.069
*Splenectomy, n (%)*	3 (9.7)	3 (14.3)	0.675
Antibody removal before KTx^b^, n (%)	15 (48.4)	21 (100.0)	<0.001
POD at diagnosis of AABMR, median (range)	N/A	5 (0–19)	N/A

ABOi, ABO-incompatible; KTx, kidney transplantation; AABMR, acute antibody mediated rejection; HLA, human leukocyte antigen; WIT, worm ischemic time; TIT, total ischemic time; FK, tacrolimus; CyA, cyclosporine A; MMF, mycophenolate mofeti; AZ, azathioprine; CPA, cyclophosphamide; POD, post-operative days; N/A, not applicable.

a Average number of HLA mismatches for each recipient.

b The number of patients who received antibody removal before KTx.

### Prediction of AABMR After ABOi KTx Using anti-A and B Abs by the CD31-ABO Microarray

**FIGURE 4:**
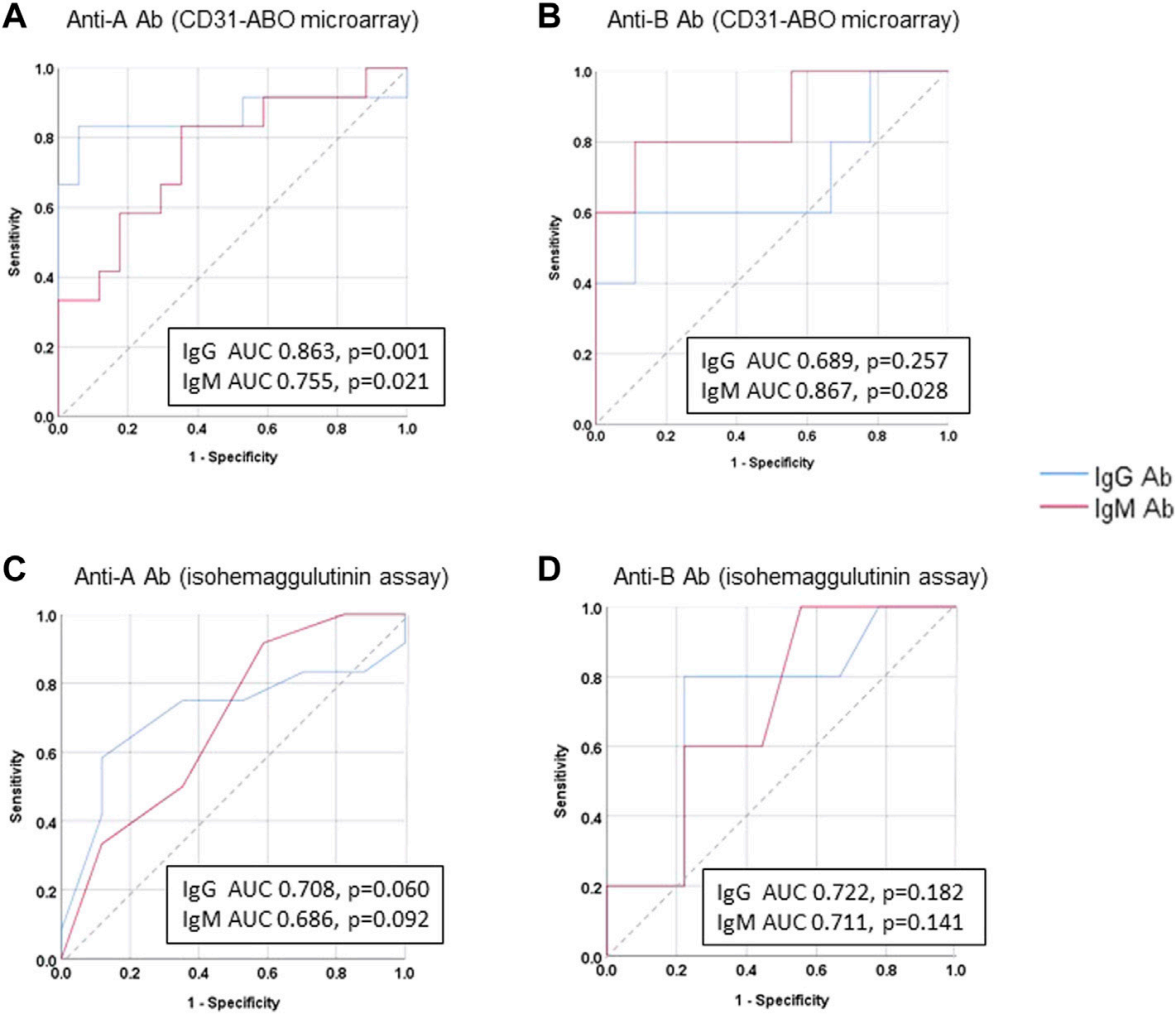
Receiver operating characteristics (ROC) curve analysis for predicting AABMR based on initial anti-A and B Ab. **(A)** anti-A Ab in CD31-ABO microarray, **(B)** anti-B Ab CD31-ABO microarray, **(C)**: anti-A Ab in isohemaggulutinin method, **(D)** anti-B Ab in isohemaggulutinin method.

**FIGURE 5:**
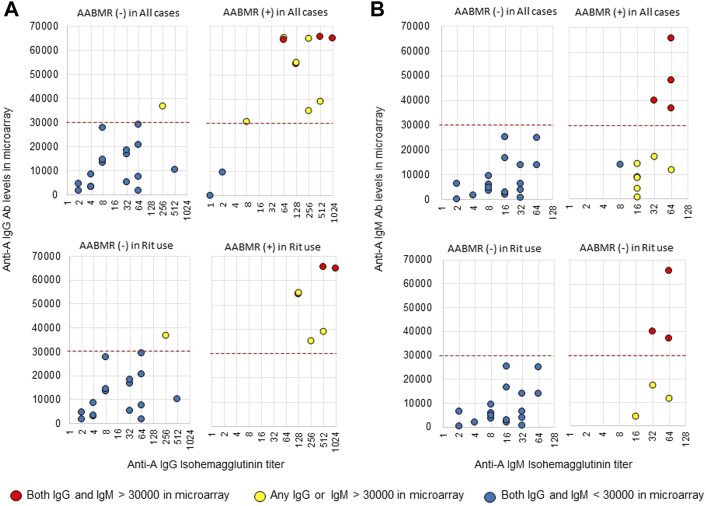
Comparison of anti-A IgG and IgM Abs between the isohemagglutinin and CD31-ABO microarray methods in blood group A-incompatible KTx patients. All samples were collected before desensitization therapy for ABOi KTx. **(A)** Anti-A IgG Ab. **(B)** Anti-A IgM Ab. Upper and lower figures are the results from all patients and rituximab (Rit)-used patients, respectively. Red circles are the results in patients who had both IgG and IgM antibody levels >30000 in the CD31-ABO microarray. Yellow circles are the results in patients who had any IgG or IgM antibody levels >30000 in the CD31-ABO microarray. Blue circles are the results in patients who had both IgG and IgM antibody levels <30000 in the CD31-ABO microarray. The x-axis is isohemagglutinin titers on a log2 scale; titer values of zero are displayed as 1 on the graph. The y-axis is antibody level on the CD31-ABO microarray. AABMR, acute antibody-mediated rejection.

**FIGURE 6:**
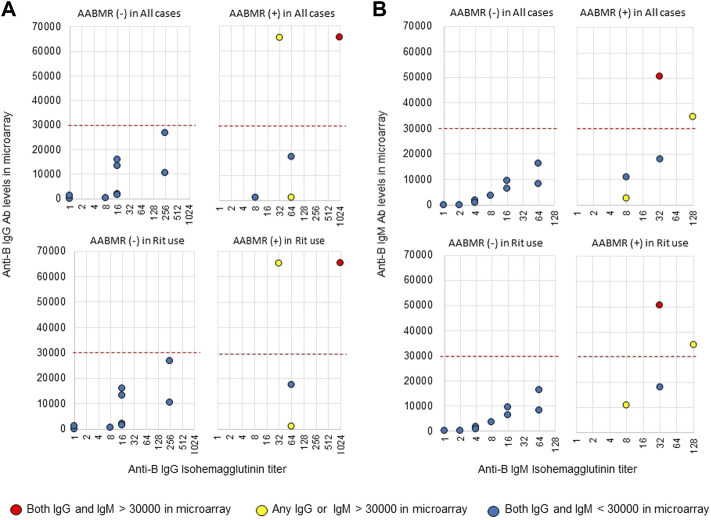
Comparison of anti-B IgG and IgM Abs between the isohemagglutinin and CD31-ABO microarray methods in blood group B-incompatible KTx patients. All samples were collected before desensitization therapy for ABOi KTx. **(A)** Anti-B IgG Ab. **(B)** Anti-B IgM Ab. Upper and lower figures are the results from all patients and rituximab (Rit)-used patients, respectively. Red circles are the results in patients who had both IgG and IgM antibody levels >30000 in the CD31-ABO microarray. Yellow circles are the results in patients who had any IgG or IgM antibody levels >30000 in the CD31-ABO microarray. Blue circles are the results in patients who had both of IgG and IgM antibody levels <30000 in the CD31-ABO microarray. The x-axis is isohemagglutinin titers on a log2 scale; titer values of zero are displayed as 1 on the graph. The y-axis is antibody level on the CD31-ABO microarray. AABMR, acute antibody-mediated rejection.

The area under the receiver operating characteristic (ROC) curve (AUC) indicated the significant prognostic power for AABMR after ABOi KTx using initial Ab levels measured by the CD31-ABO microarray, except for anti-B IgG Ab (Figures 4A,B). The prognostic power in the CD31-ABO array was better than those of isohemaggulutinin assay (Figures 4C,D). Table 4 shows the comparison of the prognostic power for AABMR with several cut-offs, suggesting the CD31-ABO microarray had higher prognostic power for AABMR than isohemagglutinin method. Any initial IgG or IgM Ab levels against donor blood type >30,000 in the CD31-ABO microarray showed high sensitivity, specificity, positive predictive value (PPV), and negative predictive value (NPV) in both anti-A and anti-B Abs. After excluding the patients whose rituximab was not used, these significant results could be seen in rituximab-based protocol patients (Table 4). To investigate whether Ab levels in the CD31-ABO microarray would more accurately predict AABMR after ABOi KTx than isohemagglutinin method, initial anti-A and B Abs of the samples obtained before desensitization therapy were compared (Upper Figures 5, 6). In A-incompatible KTx, anti-A IgG Ab levels by microarray were significantly higher in the AABMR (+) group than those in the AABMR (-) group (median: 54721 vs. 10211, *p* < 0.001). Ten out of 12 patients with AABMR (83.3%) had anti-A IgG Ab levels >30,000 in the CD31-ABO microarray; in contrast, only 1 out of 17 patients without AABMR (5.9%) had anti-A IgG Ab levels >30,000 (upper Figure 5A). Anti-A IgM Ab levels in the CD31-ABO microarray were significantly higher in the AABMR (+) group than those in the AABMR (-) group (median: 14277.5 vs. 5887, *p* = 0.03). No one had anti-A IgM Ab levels >30,000 in the AABMR (-) group, in contrast, 4 out of 12 patients with AABMR had anti-A IgM Ab levels >30,000 in the microarray (upper Figure 5B). Eight out of 12 patients with AABMR had anti-A IgM Ab levels <30,000 in the CD31-ABO microarray. However, six of these samples had anti-A IgG Ab levels >30,000 by microarray; probably, these anti-A IgG Abs induced AABMR in these patients (yellow circles in upper Figure 5B). Taken together, 10 out of 12 patients with AABMR (83.3%) had initial anti-A IgG or IgM Ab levels >30,000 in A-incompatible KTx, as shown by the CD31-ABO microarray. When we analyzed the predictive value of AABMR in the rituximab-based protocol patients, 6 out of 6 patients (100%) had anti-A IgG or IgM Ab levels >30,000 in A-incompatible KTx (Lower Figures 5A,B). Figure 6 shows anti-B Abs in patients undergoing B-incompatible KTx. Anti-B IgG Ab levels in the CD31-ABO microarray were significantly higher in the AABMR (+) group than those in the AABMR (-) group (median: 16378 vs. 1970, *p* = 0.047). Anti-B IgM Ab levels in the CD31-ABO microarray were significantly higher in the AABMR (+) group than those in the AABMR (-) group (median: 18058 vs. 3481, *p* = 0.021). No one had anti-B IgG and IgM Ab levels >30,000 using the CD31-ABO microarray in the AABMR (-) group (Upper Figures 6A,B). Three out of 5 patients with AABMR (60.0%) had initial anti-B IgG or IgM Ab levels >30,000 in B-incompatible KTx, as shown by the CD31-ABO microarray. When we analyzed the predictive value of AABMR in the rituximab-based protocol patients, 3 out of 4 patients (75%) had anti-B IgG or IgM Ab levels >30,000 in B-incompatible KTx (Lower Figures 6A,B).

**TABLE 4:** Comparison of sensitivity, specificity, positive predictive value (PPV), negative predictive value (NPV) of AABMR after ABOi KT.

Initial Ab titers or levels against donor blood type	Sensitivity (%)	Specificity (%)	PPV	NPV
Anti-A Ab (any of IgG or IgM) in all cases				
≧16 folds by isohemagglutinin	91.7	17.7	44.0	75.0
≧32 folds by isohemagglutinin	75.0	35.3	45.0	66.7
≧64 folds by isohemagglutinin	75.0	58.8	56.3	76.9
≧15,000 by microarray	83.3	52.9	55.6	81.8
≧30,000 by microarray	83.3	94.1	90.9	88.9
Anti-B Ab (any of IgG or IgM) in All cases				
≧16 folds by isohemagglutinin	80.0	33.3	40.0	75.0
≧32 folds by isohemagglutinin	80.0	66.7	57.1	85.7
≧64 folds by isohemagglutinin	60.0	66.7	75.0	66.7
≧15,000 by microarray	80.0	66.7	57.1	85.7
≧30,000 by microarray	60.0	100.0	100.0	81.8
Anti-A Ab (any of IgG or IgM) in Rituximab-use patients				
≧16 folds by isohemagglutinin	100	17.7	35.3	100
≧32 folds by isohemagglutinin	100	35.3	54.6	100
≧64 folds by isohemagglutinin	100	58.8	46.2	100
≧15,000 by microarray	100	52.9	42.9	100
≧30,000 by microarray	100	94.1	85.7	100
Anti-B Ab (any of IgG or IgM) in Rituximab-use patients				
≧16 folds by isohemagglutinin	100	33.3	40.0	100
≧32 folds by isohemagglutinin	100	66.7	57.1	100
≧64 folds by isohemagglutinin	75	66.7	57.1	100
≧15,000 by microarray	100	66.7	57.1	100
≧30,000 by microarray	75	100	100	90.0

Samples obtained after ABOi KTx were also investigated (Supplementary Figures S7, S8). The timing of plasma sample collection was different in each case. In patients without AABMR, the samples were collected within 1 month after ABOi KTx. Plasma samples were collected when AABMR was clinically suspected, but before treatment. Four out of 16 cases (25%) had anti-A IgG or IgM Abs >30,000 in A-incompatible KTx, as shown by the CD31-ABO microarray (Supplementary Figure S7). One out of 5 cases (20%) had anti-B IgG or IgM Abs >30,000 in B-incompatible KTx, as shown by the CD31-ABO microarray (Supplementary Figure S8). However, there were no significant differences between the AABMR (+) and AABMR (-) groups in levels of anti-A and -B Abs examined by both the isohemagglutinin and CD31-ABO microarray methods. We showed how Ab levels changed before and after ABOi KTx in Supplementary Figure S9. Ab titers by isohemagglutinin method before desensitization were not significantly different between AABMR (+) and AABMR (-). However, CD31-ABO microarray could show that they were significantly higher in AABMR (+) than ABMR (-) before desensitization therapy. As described above, Ab titers after ABOi KTx were not significantly different between AABMR (+) and AABMR (-) in either of the two methods.

## Discussion

To evaluate the risk of AABMR in patients undergoing ABOi transplants, anti-A or -B Ab titers are required. There are several methods to measure anti-A and -B Ab titers, such as the tube test assay (17), the column agglutination technique (18), flow cytometry (19, 20), and the solid phase red cell adherence technique (21). In these methods, the reaction of Abs against RBCs is used to determine anti-A or -B Ab titers. ABO blood group antigens are expressed on both RBCs and KECs. RBCs are used as targets to investigate anti-A or -B Ab titers from the convenience of use and obtainability before ABOi KTx. Initial anti-A or -B Ab titers against RBCs are a good predictor of AABMR in ABOi KTx (22), suggesting ABO blood group antigens are similar between RBCs and KECs. However, CD31 is major protein linked to ABO carbohydrate antigens in human KECs, and is different from those expressed on RBCs (9). Anti-blood group Ab epitopes against ABO blood group antigens are thought to be different between RBCs and endothelial cells (10). The Ab removal-free protocol has been reported in ABOi KTx when anti-A or -B Ab titers are below 64-fold, resulting in no AABMR (23). In contrast, anti-A or -B Ab induced AABMR and thrombotic microangiopathy in ABOi-KTx remain critical issues (24), and heavier immunosuppression is required. To clarify the risk of AABMR and avoid infectious events due to over immunosuppression after ABOi KTx, the real reaction of anti-A or -B Ab against ABO blood group antigens on KECs needs to be known.

In the present study, a method to evaluate anti-A and -B Abs that react to ABO blood group antigens expressed on KECs was developed. rCD31 proteins containing ABO carbohydrate antigens were used to form the CD31-ABO microarray. ABO glycans were compared between rCD31 used for the CD31-ABO microarray and CD31 derived from normal human kidney by MS analysis, which suggested that the CD31-ABO microarray was a mimic of ABO blood group antigens on human KECs. Anti-A and -B Abs titers were roughly correlated between the isohemagglutinin and CD31-ABO microarray methods. However, there was great variability in anti-A and -B Abs levels in the CD31-ABO microarray among patients who had the same Ab titer using the isohemagglutinin method. The desensitization therapy contents were not significantly different between the two groups of patients with and without AABMR, except for Ab removal. In spite of isohemagglutinin Ab titers using RBCs that were not significantly different between the two groups, the patients who suffered from AABMR had significantly higher Ab levels of the CD31-ABO microarray in AABMR (+). The sensitivity of predicting AABMR in the CD31-ABO microarray was not high in B-incompatible KTx when the cut-off Ab level was >30,000. However, there were no patients who had anti-B Ab levels >30,000 in B-incompatible KTx without AABMR, using the CD31-ABO microarray (the specificity of predicting AABMR was 100%). In this study, we found that Ab levels measured by the CD31-ABO microarray was the most important to predict AABMR after ABOi-KTx.

Ab levels examined by the CD31-ABO microarray were low in the samples obtained when AABMR was clinically suspected. After ABOi KTx, it is possible that anti-A or -B Ab reacted to ABO antigens on graft endothelial cells and were absorbed. The absorption of anti-A or -B Abs could affect plasma Ab levels determined using the CD31-ABO microarray more than the isohemagglutinin method because of the specificity to KECs. Thus, the CD31-ABO microarray might not be a significant tool to predict AABMR after ABOi KTx.

There are limitations to the present study. We do not routinely examine blood group A subtype. However, 99.8% of Japanese people of blood type A belong to A1 (25, 26). The cohort of ABOi KTx consisted of a heterogeneous population who received different immunosuppressive protocols in this study. However, the desensitization therapy protocol for ABOi KTx varies from institution to institution. In a real situation for ABOi KTx, we tried to examine the new method of CD31-ABO microarray on various patients in the present study. The number of samples obtained from patients with ABOi KTx was small, especially B-incompatible KTx. To elucidate the value of the CD31-ABO microarray to predict AABMR in ABOi KTx, further examination using more samples is required. Samples from the day of the ABOi KTx were not stored and could not be investigated by CD31-ABO microarray. It is important to know Ab levels that should be decreased by desensitization therapy before ABOi KTx. A multi-center study using the CD31-ABO microarray is currently ongoing to determine if AABMR may be avoided after ABOi KTx and how much high Ab levels should be decreased before ABOi KTx.

In conclusion, a novel method to investigate anti-A and -B Abs was developed, which were mimics of ABO blood group antigens on KECs. This may identify the precise risks of AABMR after ABOi KTx in advance. As large meta-analysis of ABOi KTx has shown, graft and patient survival in ABOi KTx were significantly worse than those of ABOc KTx (6, 7). They suggest two issues of ABOi KTx: AABMR and infectious events. According to the results of the CD31-ABO microarray, we will be able to strengthen or reduce desensitization therapy, resulting in decreased numbers of AABMR and infectious events.

## Data Availability

The raw data supporting the conclusion of this article will be made available by the authors, without undue reservation.

## Supplementary Material

Supplementary Figure S1 Accumulated mass spectra of glycopeptides composed of a core peptide (VLENSTK including Asn-453) derived from recombinant CD31. Spectrum for **(A)** HEK293H, **(B)** for HEK293A, **(C)** for HEK293B. Glycan compositions (the numbers of Hex, HexNAc, and Fuc, on trimannosyl core) characteristic or increased against H are presented in each spectrum with triangles, and those containing multiple Fuc are in blue. Assignment of signals are summarized in Supplementary Table S1.

Supplementary Figure S2 Accumulated mass spectra of glycopeptides composed of a core peptide (EGKPFYQMTSNATQAFWTK including Asn-551) derived from recombinant CD31. Spectrum for **(A)** HEK293H, **(B)** for HEK293A, **(C)** for HEK293B. Glycan compositions (the numbers of Hex, HexNAc, and Fuc on trimannosyl core) characteristic or increased against H are presented in each spectrum with triangles, and those containing multiple Fuc are in blue. Assignment of signals are summarized in Supplementary Table S2. Presumed addition of GalNAc and Gal on H antigen are shown with arrow and predicted glycan structures are shown.

Supplementary Figure S3 Accumulated mass spectra containing human kidney CD31 glycopeptides; VLENSTK including Asn-453. **(A)** Spectrum for blood type O, **(B)** for blood type A, **(C)**, for blood type B. Signals indicated with glycan compositions (the numbers of Hex, HexNAc, and Fuc on trimannosyl core) are assigned to CD31 peptide having various glycans. MS/MS spectra were compared for signals shown with red triangle (Supplementary Figures S4–S6).

Supplementary Figure S4 MS/MS spectra of glycopeptides derived from the normal parts of human kidney CD31 (type O). MS/MS spectra of glycopeptides assigned as compositions 221, 222, and 332, are compared. The core peptide of selected glycopeptides is common and found to be VLENSTK, from core peptide-related ions, Y0, Y1, and Y2, as indicated with green arrow heads. Y1+F (fucose) signals indicating the attached glycan has core-fucose are shown with orange arrow heads. Glycan-derived fragment ions are indicated with blue arrow heads. One (or two) putative glycan structures is shown on the right side of each spectrum.

Supplementary Figure S5 MS/MS spectra of glycopeptides derived from the normal parts of human kidney CD31 (type A). MS/MS spectra of selected glycopeptides are compared. The core peptide of the selected glycopeptides is common and found to be the same for Supplementary Figure S4, VLENSTK, from core peptide-related ions, Y0, Y1, and Y2. Y1+F (fucose) signals indicating the attached glycan has core-fucose are shown with orange arrow heads. Glycan-derived fragment ions are indicated with blue arrow heads. One (or two) putative glycan structure is shown on the right side of each spectrum.

Supplementary Figure S6 MS/MS spectra of glycopeptides derived from the normal parts of human kidney CD31 (type B). MS/MS spectra of selected glycopeptides are compared. The core peptide of the selected glycopeptides is common and found to be the same for Supplementary Figures S4, S5, VLENSTK, from core peptide-related ions, Y0, Y1, and Y2. Y1+F (fucose) signals indicating the attached glycan has core-fucose are shown with orange arrow heads. Glycan-derived fragment ions are indicated with blue arrow heads. One (or two) of putative glycan structure is shown on the right side of each spectrum.

Supplementary Figure S7 Comparison of anti-A IgG and IgM Abs between the isohemagglutinin and CD31-ABO microarray methods in blood group A-incompatible KTx patients. All samples were collected after ABOi KTx. In patients with AABMR (+), the plasma samples were collected when AABMR was clinically suspected, but before treatment. **(A)**: Anti-A IgG Ab. **(B)**: Anti-A IgM Ab. Red circles are the results in patients who had both IgG and IgM antibody levels > 30000 in the CD31-ABO microarray. Yellow circles are the results in patients who had any IgG and IgM antibody levels > 30000 in the CD31-ABO microarray. Blue circles are the results in patients who had both IgG and IgM antibody levels < 30000 in the CD31-ABO microarray. The x-axis is isohemagglutinin titers on a log2 scale; titer values of zero are displayed as 1 on the graph. The y-axis is antibody level on the CD31-ABO microarray. AABMR: acute antibody-mediated rejection.

Supplementary Figure S8 Comparison of anti-B IgG and IgM Abs between the isohemagglutinin and CD31-ABO microarray methods in blood group B-incompatible KTx patients. All samples were collected after ABOi KTx. In patients with AABMR (+), the plasma samples were collected when AABMR was clinically suspected, but before treatment. **(A)**: Anti-B IgG Ab. **(B)**: Anti-B IgM Ab. Blue circles are the results in patients who had both IgG and IgM antibody levels < 30000 in the CD31-ABO microarray. The x-axis is isohemagglutinin titers on a log2 scale; titer values of zero are displayed as 1 on the graph. The y-axis is antibody level on the CD31-ABO microarray. AABMR, acute antibody-mediated rejection.

Supplementary Figure S9 Ab titers’ changes before and after ABOi KTx. **(A)**: anti-A Ab titer measured by isohemaggulutinin assay, **(B)**: anti-B Ab titer measured by isohemaggulutinin assay, **(C)**: anti-A Ab level measured by CD31-ABO microarray, **(D)**: anti-B Ab level measured by CD31-ABO microarray. pre: before desensitization therapy, Tx: on the day of transplantation (Ab levels weren’t examined at this point by CD31-ABO microarray), post: on the day of suspicious AABMR [the samples were collected within 1 month after ABOi KTx in the patients of AABMR(-)]. The y-axis is isohemagglutinin titers on a log2 scale; titer values of zero are displayed as 1 on the graph **(A,B)**. The y-axis is antibody level on the CD31-ABO microarray **(C,D)**. AABMR, acute antibody-mediated rejection. ^∗^ *p* < 0.05.

## Abbreviations

AABMR, acute antibody-mediated rejection; ABOi, ABO-incompatible; ABOc, ABO-compatible; HEK, human embryonic kidney; KEC, kidney endothelial cell; KTx, kidney transplantation; MS, mass spectrometry; NPV, negative predictive value; PPV, positive predictive value; RBC, red blood cell; rCD31, recombinant CD31 protein; ROC, receiver operating characteristic.
