# Supplementary figures and images for: A Novel Method of CD31-Combined ABO Carbohydrate Antigen Microarray Predicts Acute Antibody-Mediated Rejection in ABO-Incompatible Kidney Transplantation
Source: Transpl Int. 2022 Mar 23;35:10248. doi: 10.3389/ti.2022.10248 (PMC8985549; doi:10.3389/ti.2022.10248)

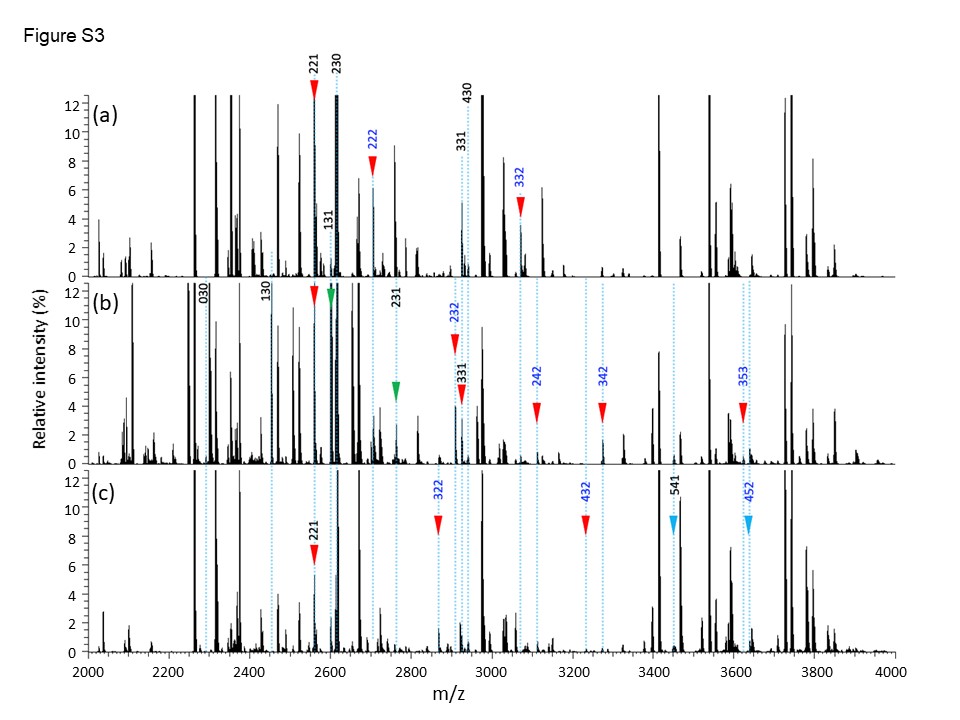

Supplement: Supplementary file 2 [file Image3.JPEG]

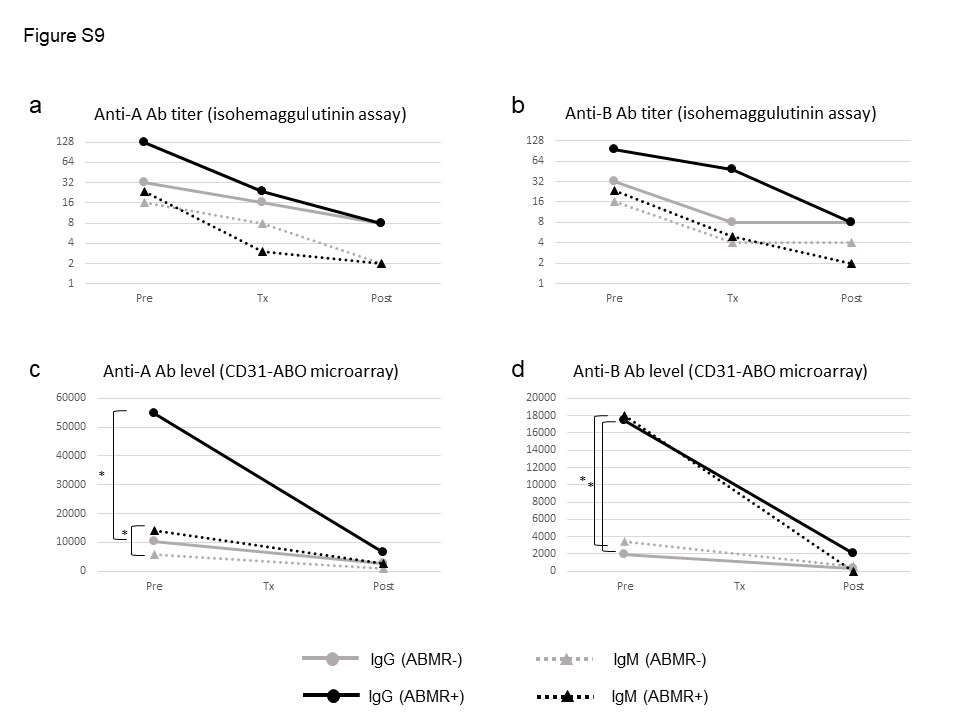

Supplement: Supplementary file 3 [file Image9.JPEG]

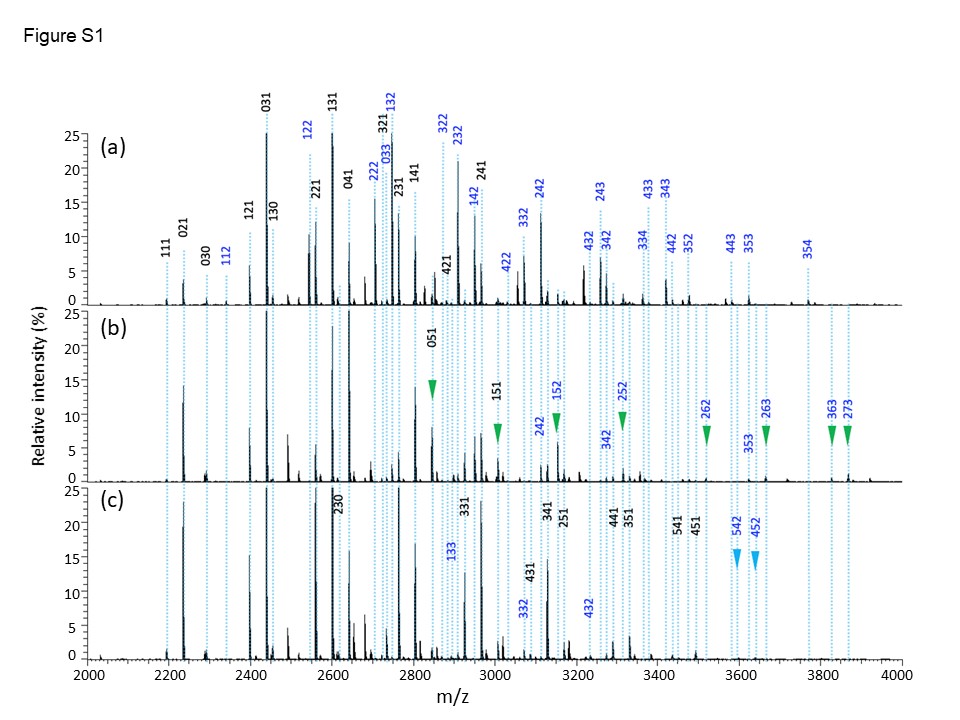

Supplement: Supplementary file 4 [file Image1.JPEG]

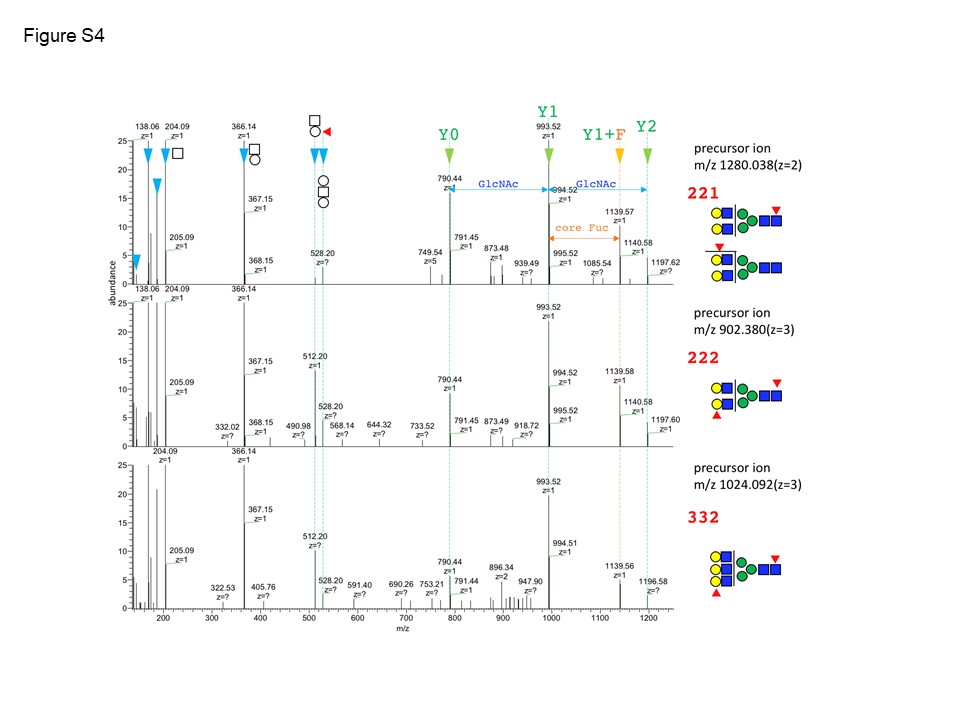

Supplement: Supplementary file 5 [file Image4.JPEG]

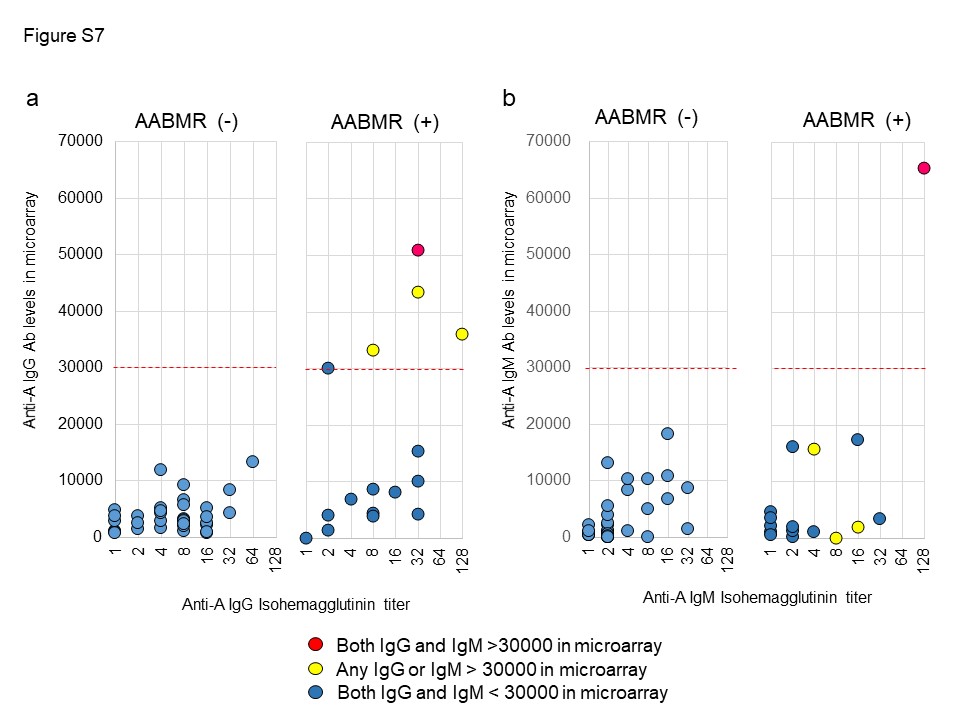

Supplement: Supplementary file 6 [file Image7.JPEG]

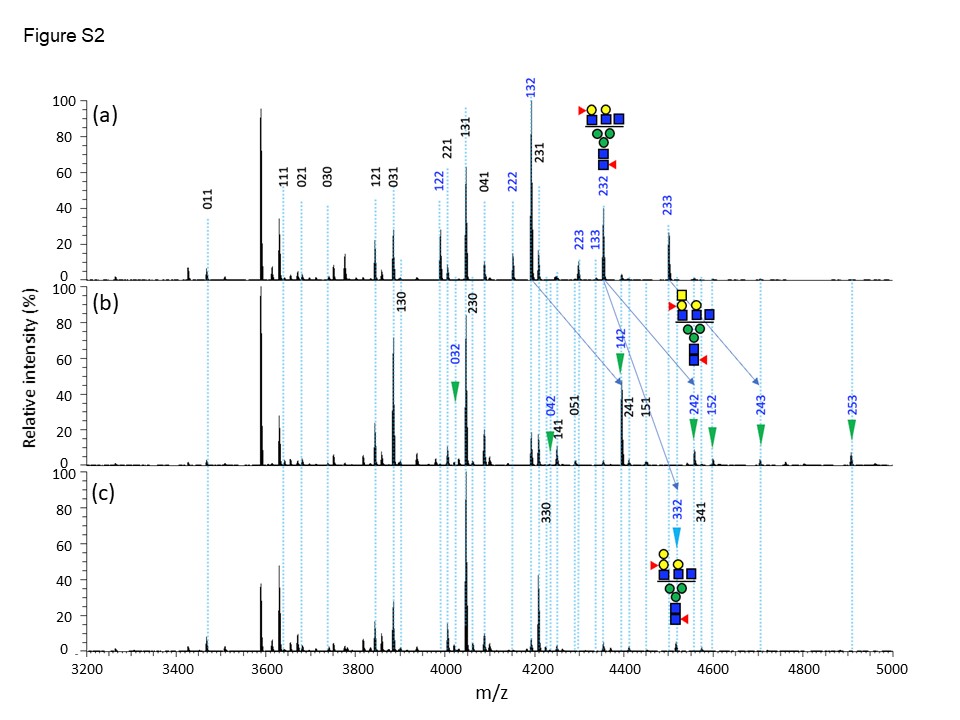

Supplement: Supplementary file 7 [file Image2.JPEG]

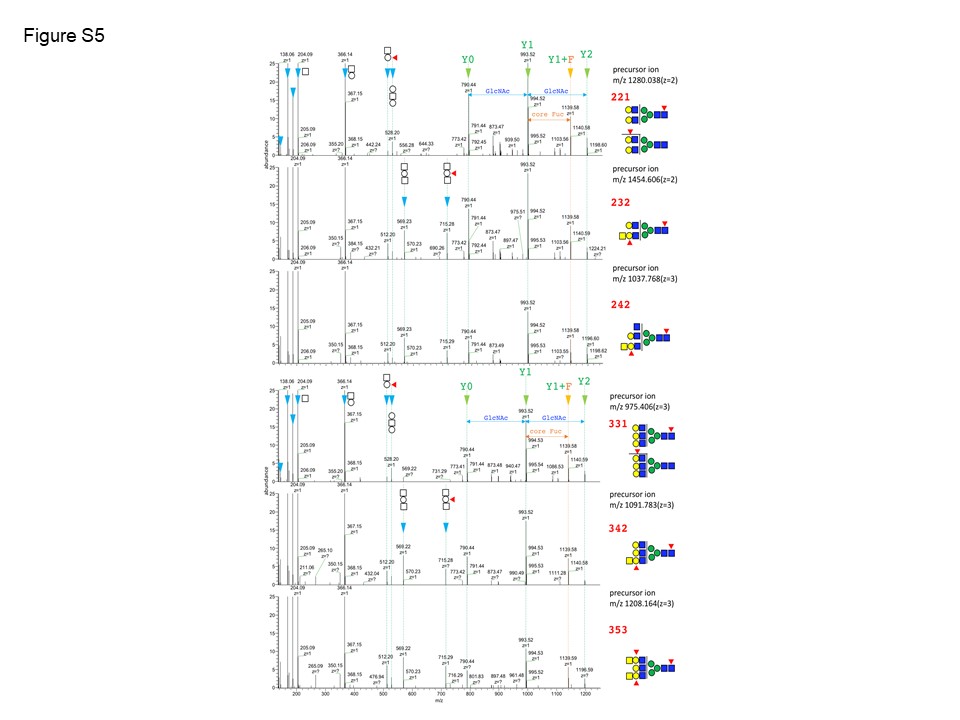

Supplement: Supplementary file 8 [file Image5.JPEG]

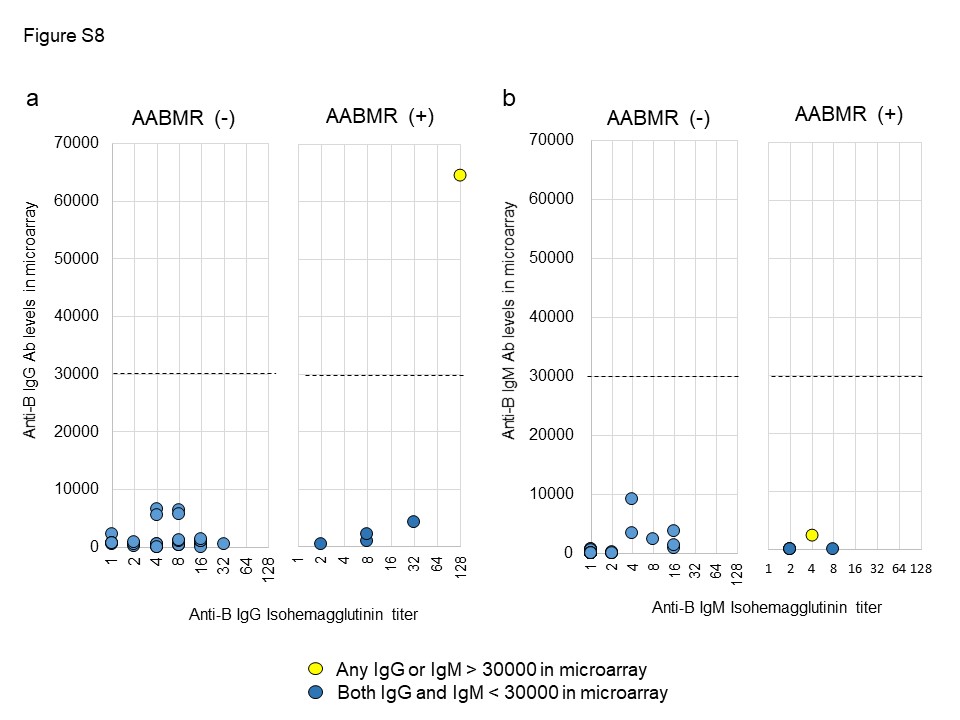

Supplement: Supplementary file 10 [file Image8.JPEG]

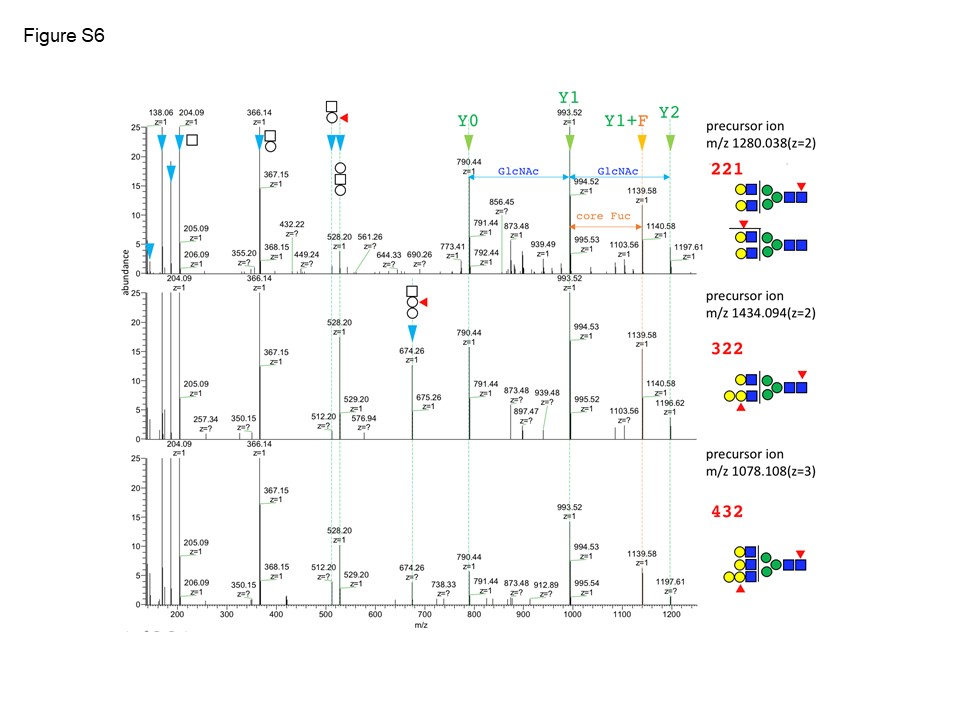

Supplement: Supplementary file 11 [file Image6.JPEG]
